# Replisomal coupling between the α-pol III core and the τ-subunit of the clamp loader complex (CLC) are essential for genomic integrity in *Escherichia coli*

**DOI:** 10.1016/j.jbc.2025.108177

**Published:** 2025-01-10

**Authors:** Lauren J. Butterworth, Malisha U. Welikala, Cody W. Klatt, Kaitlyn E. Rheney, Michael A. Trakselis

**Affiliations:** Department of Chemistry and Biochemistry, Baylor University, Waco, Texas, USA

**Keywords:** *E. coli*, coupling, decoupling, alpha (α), Pol III core, Pol C, polymerase III holoenzyme, tau (τ), clamp loader complex (CLC), unwinding, DNA replication, genomic instability, replisome

## Abstract

Coupling interactions between the alpha (α) subunit of the polymerase III core (α-Pol III core) and the tau (τ) subunit of the clamp loader complex (τ-CLC) are vital for efficient and rapid DNA replication in *Escherichia coli*. Specific and targeted mutations in the C-terminal τ-interaction region of the Pol III α-subunit disrupted efficient coupled rolling circle DNA synthesis *in vitro* and caused significant genomic defects in CRISPR-Cas9 *dnaE* edited cell strains. These α-Pol III mutations eliminated the interaction with τ-CLC but retained WT polymerase and exonuclease activities. The most severely affected mutant strains, *dnaE*:Y1119A and *dnaE*:L1097/8S, had significantly reduced doubling times, reduced fitness, and increased cellular length phenotypes as a result of this targeted decoupling of the replisome and the generation of replication stress. Those strains also showed significant SOS induction from unwound but unreplicated regions within the genome. In support, significant ssDNA gaps were detected by fluorescence microscopy and quantified by fluorescence activated cytometry using an *in situ* PLUG assay for those *dnaE*:mut strains. By comparing the biochemical and genomic consequences of disrupting the τ-CLC-α-Pol III coupling contacts, we have unveiled a more cohesive picture and mechanistic understanding of replisome dynamics and the essential interactions required to maintain overall fitness through a stable genome.

The interactions and integration of enzymes within a replisome complex are required to efficiently synthesize DNA in all forms of life (1). The *Escherichia coli* replisome is comprised of a minimal assembly of 13 proteins working together to duplicate parental DNA strands into daughter strands (2). The roles of each of these enzymes are well-established, and the dynamic behavior of this network of enzymes, revealed through single molecule studies (3), has paved the way toward a greater understanding of the specific inner workings of replisome plasticity. This dynamic regulation is beneficial in overcoming obstacles during DNA replication to maintain genomic integrity (4). However, there are many aspects of this stochastic replisomal symphony that are not well understood, including how individual proteins communicate and regulate their activities to replicate DNA efficiently.

Enzymes at the forefront of the replication fork serve to control the rate of replication, ensuring that all enzymatic activities are in equilibrium. At the leading edge of the replisome, the homohexameric DnaB helicase unwinds duplex DNA, whose mechanics and regulation continue to be extensively explored (5, 6, 7, 8). DnaB anchors itself to the fork junction, creating a divide between leading and lagging strand synthesis, effectively establishing the replication fork. This allows concurrent binding of the polymerase III holoenzyme and DNA primase (DnaG) (9), where they can coordinate the synthesis of ribonucleotide primers that are extended into new leading and lagging daughter strands. Meanwhile, the clamp loader complex (CLC) facilitates the processivity of DNA synthesis by loading and unloading the beta (β) sliding clamp onto parent strands (10) and directing the recruitment and retention of the Pol III core. CLC also provides for communication between the Pol III core and DnaB (Fig. 1). Specifically, the τ-subunit of the CLC directly connects the catalytic α-subunit of the Pol III core to the DnaB helicase through its C-terminal linker to coordinate DNA synthesis and unwinding. Though these individual enzymatic roles are well-established, the specific mechanics governing enzymatic coupling within a larger replisome complex necessary for proper genome maintenance are not.

**Figure 1:**
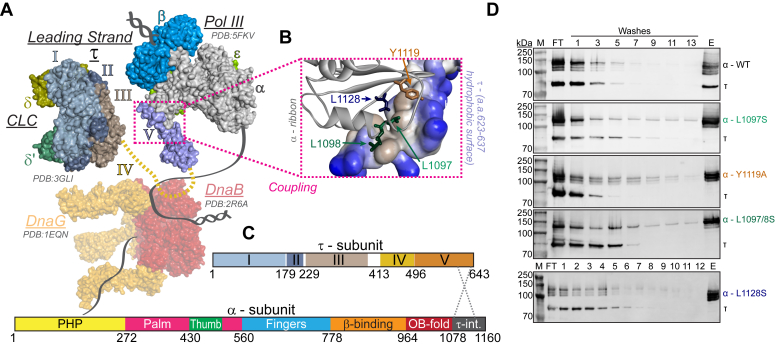
**Molecular model of the *E. coli* replisome hub for unwinding and synthesis.***A*, X-ray crystal structures of DnaB (PDBID: 2R6A, *red*), DnaG (PDBID: 1EQN, *orange*), Pol III-β-τ (PDBID: 5FKV, α-*gray*, ε-*green*, θ-*gray*; β, *blue*; τ; *lilac*,), and γ-CLC (PDBID: 3GLI, domains I, *light steel blue*, II, *Bermuda gray*, III, *akaroa*) were used to model a proposed leading strand bacterial replisome that physically couples unwinding and synthesis. Unstructured domain IV of τ is shown as a *dashed line* (*mustard*). *B*, a close-up of the proposed coupling interaction of the C*-*terminus of α interacting with a hydrophobic surface representation of domain V of τ. *C*, linear domain structures of τ and α, highlighting the interaction site (τ-int) with *dashed lines*. *D*, His-Pol-α pull-downs with indicated mutations interacting with τ as probed by Western blot analysis with specific antibodies. M-molecular weight markers; FT-flow through; 12 to 13 washes indicated; E−elution with imidazole. CLC, clamp loader complex.

Coupling between enzymatic activities is accomplished through communication of interfacial amino acid residues between interacting proteins (Fig. 1). If these connections are severed, then the replisome becomes decoupled. If decoupling persists, replisome coordination is reduced, leading to labile ssDNA accumulation. For example, if Pol III pauses to allow for translesion synthesis or repriming, a lack of coordination allows the helicase to continue, leaving excess ssDNA exposed and creating genomic instability (11). However, if the helicase slows to allow for Pol III pausing, when in proper contact with τ, then these enzymes can easily recouple their activities. Thus, the replisome has evolved to become more dynamic to help prevent labile ssDNA from forming or allow for reduced activity upon encountering genomic obstacles including DNA damage, alternative DNA structures, or stably bound proteins. With protein contacts transiently breaking and reforming to continuously adapt to these obstacles encountered by the replisome, it guarantees that the genome is protected (12). However, if the connection with a stalled polymerase cannot be reestablished, the helicase will continue unwinding, exposing vital and susceptible genomic ssDNA stretches. When long stretches of ssDNA are exposed, bacteria have evolved a transcription checkpoint response, termed the SOS response, to compensate for perceived genomic insults (13). The SOS response is a mechanism adapted by *E. coli* to upregulate genes necessary to handle cellular stress and repair damaged DNA. However, one consequence of the SOS response is activation of translesion DNA polymerases, *i.e.* Pol V, that are more error-prone, contributing to genomic mutations (14).

Polymerases are replicative enzymes that synthesize new strands of DNA during elongation. These enzymes must replicate with high speed and accuracy to maintain genomic integrity. The *E. coli* Pol III core holoenzyme contains three protein subunits. The largest is the α-subunit (132 kDa), encoded by the *dnaE* gene, containing the polymerase catalytic active site (15). The epsilon (ε) subunit (27 kDa) contains the proofreading exonuclease activity. The smallest subunit, theta (θ) (10 kDa), is of unknown function, but evidence suggests that it aids in the stability of the heterotrimeric core (15, 16). The CLC clamp loader is quite a complex enzyme both structurally and functionally. The CLC is a seven-subunit complex, where its composition is τ_n_γ_3-n_δδ′, where n ranges from 1 to 3 combination of τ or γ subunits. The same gene, *dnaX*, encodes the τ and γ subunits, but τ (71 kDa) is full length, and γ (47 kDa) is truncated as the result of translational frameshift (17). Both are catalytic components containing DNA-dependent ATPase activity (18). CLC forms a pentameric ring that includes ATPase activity to load the β-sliding clamp onto DNA using energy from ATP hydrolysis (19, 20). The χ and ψ subunits form a heterodimer attached to the pentameric ring. This heterodimer moderates interactions between DNA single-stranded binding proteins (SSB) and CLC (21).

The τ-subunit of the CLC, often referred to as the central organizer (22), is responsible for coordinating unwinding and synthesis (23, 24) by acting as a functional link. Certain τ domains are connected through flexible linkers, which act as sites of interaction, providing greater flexibility and mobility for τ to interact with DnaB and α-subunits at the replication fork (25). In fact, when τ is absent, replisome unwinding and synthesis move at a slower rate (23). Interestingly, each individual τ-subunit can bind to an α-subunit of Pol III core (26). Therefore, when CLC consists of two τ-subunits (τ_2_γ_1_δδ′ χψ), it can bind and interact with two α-subunits, thus coupling to both the leading and lagging strand polymerases (26) to DnaB (24). When three τ-subunits (τ_3_δδ′ χψ) are present as the CLC within the replisome, it can bind up to three polymerases. This is especially useful when performing its duties of switching a β-clamp from a complete Okazaki fragment to a new fragment, as it allows the third polymerase to act in reserve for lagging strand synthesis to maintain similar speeds to that of leading strand synthesis (27). Therefore, τ and α play significant roles in coordinating optimal replisome function through direct contacts thus enzymatic coupling is essential for maintaining genomic and cellular integrity (15).

To provide greater insight into the biochemical importance of this coupling phenomenon, we mutated specific amino acids within the C-terminus of the Pol III α-subunit predicted to disrupt interaction with Domain V of the CLC τ -subunit (Fig. 1). The inherent enzymatic ability of these α-Pol III variants on short primer template substrates were found to be equivalent, however, when assembled into a full replisome, several of the α-Pol III mutants, Y1119A, L1097/8S, and L1128S, showed reduced DNA synthesis ability in leading strand replication assays. To correlate these *in vitro* observations with expected phenotypic decoupling *in vivo*, we made corresponding site-specific genomic mutations in the *dnaE* gene, encoding for α-Pol III, using CRISPR/Cas9 editing. Overall, the *dnaE*:L1097/8S and *dnaE*:Y1119A mutant strains displayed the most severe phenotypes, while the *dnaE*:L1128S strain was only mildly affected. Both *dnaE*:L1097/8S and *dnaE*:Y1119A displayed significantly reduced growth rates and were outcompeted by the parental stain. Unexpectedly, the *dnaE*:L1097S single mutation was outcompeted by the parental strain the quickest and may be the result of longer-term coculture conditions compared to the rest of the experiments. Both *dnaE*:L1097/8S and *dnaE*:Y1119A displayed cellular filamentation indicative of genomic stress preventing fission, prevalent ssDNA gaps, significantly increased proportion of SOS-positive cells, and increased cell death. The phenotypic effects of *dnaE*:L1128S were more muted with moderate SOS induction and more limited cell death. Altogether, we have shown that specific residues within the C-terminus of α-Pol III have significant roles in maintaining contact with τ-CLC and that these coupling interactions are essential for reducing ssDNA gap occurrence that can impact genomic integrity and cellular survival in *E. coli.*

## Results

### Pol III **α**-subunit mutations disrupt interactions with **τ**-CLC

We identified surface residues of the C-terminus of Pol III-α that interact within a hydrophobic stretch at the end of domain V of τ, and then mutated these four residues, L1097S, Y1119A, L1097/8S, and L1128S, to potentially disrupt this interaction (Fig. 1, *A*–*C*). A mutation to serine would disrupt hydrophobic interactions while keeping the amino acid side chain of the same size, while a mutation to alanine would significantly reduce the size of a bulky side chain, both disrupting communication between these subunits. The combination of the accessible surface area and electrostatic changes will be important in altering affinity, and so, Y1119A and L1097/8S were predicted to have the most deleterious effects.

Pull-down experiments were used to verify that interactions between Pol III-α and τ were disrupted with these mutations (Fig. 1*D*). The Pol III-α enzyme variants used in these experiments were designed with a 10X histidine tag on its N-terminus to bind and immobilize onto Ni^2+^ resin. Prior to performing these pull-downs, control experiments for WT α and τ-CLC confirmed that α bound well to the resin, but τ-CLC did not (Fig. S1). If an interaction remains intact, τ-CLC will bind α and coelute at high imidazole. A Western blot was used to detect the presence of either α or τ in the elution. It was clear that the WT enzymes had strong binding and coelution; however, when identical reactions were performed with the mutant Pol III-α, the τ-CLC binding was confirmed to be disrupted (Fig. 1*D*). Although the L1097S mutant had severely disrupted binding to τ, there was a faint band for τ that was just above background in the elution lane, indicating a weakened but not eliminated interaction.

### DNA synthesis is reduced for several Pol III-**α** mutants in whole replisome TFII synthesis assays

Prior to performing whole replisome assays, it was vital to show that the Pol III core enzymatic ability of each variant showed no significant difference. These mutations are far outside the catalytic active site, and so, we would not expect these mutations to affect the inherent polymerization activity. All α mutants were expressed and purified as the core Pol III complex consisting of ε and θ (Fig. S2*A*). To confirm Pol III core WT and mutant variants are equally active, we performed separate primer extension and exonuclease assays using a cyanine-5 (Cy5)-5′ end labeled 22 nt primer annealed to a 52 nt template (Table S1) and quenched at various times (Fig. S2, *A* and *B*). Experiments were performed in triplicate and electrophoresed on 20% acrylamide denaturing tris-borate-EDTA buffer sequencing gels. Bands above and below the primer represent extension or degradation, respectively (Fig. S2, *C* and *D*). Both the extension and degradation activity of the WT and mutant Pol III core enzymes were quantified and found to be consistent across all variants (Fig. S2, *E* and *F*).

**Figure 2:**
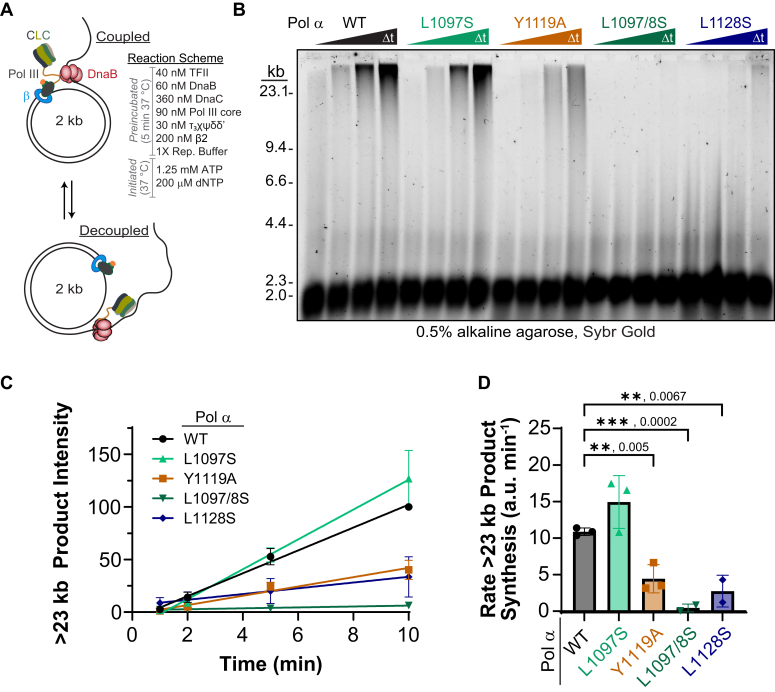
**Coupling between Pol III α and τ within the replisome is required for optimal leading strand synthesis.***A*, replisome coupling is assayed using an *in vitro* reaction scheme to assemble *Escherichia coli* proteins onto a TFII substrate and initiate unwinding and synthesis. *B*, the leading strand products were resolved on a 0.5% alkaline agarose gel stained with SYBR Gold. *C*, the linear rate of product accumulation greater than 23 kb was (*D*) quantified and plotted for three biological replicates. Error bars represent SEM, and the *black bars* indicate statistically significant differences with *p*-values indicated and represented by ∗∗<0.01 and ∗∗∗<0.001 from an unpaired two-sided *t* test. TFII, tailed form II.

To investigate DNA synthesis of these Pol III–α mutants within the context of a bacterial replisome, we assembled 12 of the *E. coli* replisome proteins onto a gapped tailed form II (TFII) substrate (Fig. S3) capable of monitoring rolling circle replication as a function of time (28). Rolling circle assays were carried out by introducing all replisomal enzymes to a reaction mixture, incubated for 5 min, initiated with dNTPs, and then quenched with EDTA at several different time points to quantify leading strand synthesis over time (Fig. 2*A*). Leading strand synthesis of the TFII is monitored both by amount and length of product stained by SYBR Gold from an agarose gel (Figs. 2*B* & S4). Interestingly, significantly lower leading strand synthesis rates for products greater than 23 kb are noted for Y1119A (2-fold), L1097/8S (>20-fold), and L1128S (4-fold) (Fig. 2, *C* and *D*). As the individual enzymatic activities of the Pol III mutants are the same (Fig. S2), any reduction in DNA synthesis can be attributed to decoupling of unwinding and polymerization controlled through contacts in the C-terminus of Pol III and the τ-subunit of the CLC.

### Genomically edited dnaE mutants limit cell growth and strain fitness

CRISPR/Cas9 *in vivo* editing was performed using a dual vector system (6) to create analogous Pol III–α mutants in the *dnaE* gene within the *E. coli* MG1655 strain. Recombination efficiency of synthetic oligonucleotides containing the desired mutation was increased by inducing λ-red genes with IPTG to increase recombination frequency after CRISPR/Cas9 targeting. Mutants were screened by novel restriction enzyme digest on colony PCR products and confirmed by sequencing targeted linear amplicons (Fig. S5). These MG1655: *dnaE*:mut strains were cured of the temperature-sensitive pREDCas9 plasmid by growth at 44 °C and pCRISPR by continuous antibiotic-selection streaking.

**Figure 3:**
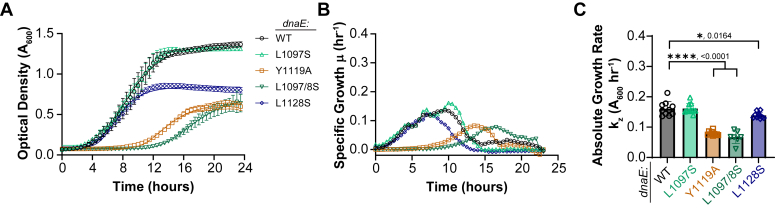
***dnaE*:mut strains show decreased growth rates in M9 minimal media.***A*, the mass doubling of each strain was monitored by *A*_*600*_ in 96-well plates. *B*, the specific growth (μ) was determined from the derivative of the curve over a rolling 30-min time. *C*, the absolute growth rate (*k*_*z*_) was determined from Equation 1 and plotted for the ten technical replicates. Error bars represent SD, and the *black bars* indicate statistically significant differences with *p*-values indicated and represented by ∗<0.05 and ∗∗∗∗<0.0001 from an unpaired two-sided *t* test.

The mass doubling times of all *dnaE*:mut strains grown in either Miller LB (Fig. S6) or M9 minimal (Fig. 3) media were monitored by *A*_600_ over 24 h at 37 °C in 96-well plates using a plate reader. The absolute growth rate (*k*_*z*_) for the WT parental MG1655 strain (0.27 h^−1^) (Fig. S6*C*) is consistent with previous studies using other instruments and strains (29, 30). Even though all strains grow fast in Miller LB, there are significant decreases in *k*_*z*_ rates *dnaE*:Y1119A, 0.22 h^−1^ and *dnaE*:L1097/8S, 0.21 h^−1^ compared to the parent (Fig. S6*C*). These differences in doubling times are amplified in M9 minimal media (Fig. 3*A*). While both the parent *dnaE*:WT and *dnaE*:L1097S strains maintain comparable but reduced growth at 0.16 h^−1^ in M9, there are significantly reduced rates for *dnaE*:Y1119A (0.08 h^−1^). *dnaE*:L1097/8S (0.07 h^−1^), and now even *dnaE*:L1128S (0.14 h^−1^) compared to the parent strain (Fig. 3, *B* and *C*). The mass doubling of *dnaE*:L1097S is not perturbed in either medium and requires the neighboring mutation of L1098S to achieve an effect, and therefore, serves as a serendipitous control for all subsequent experiments. Thus, the maximal growth rates for *E. coli* are perturbed when the binding interface between α and τ is fully disrupted.

**Figure 4:**
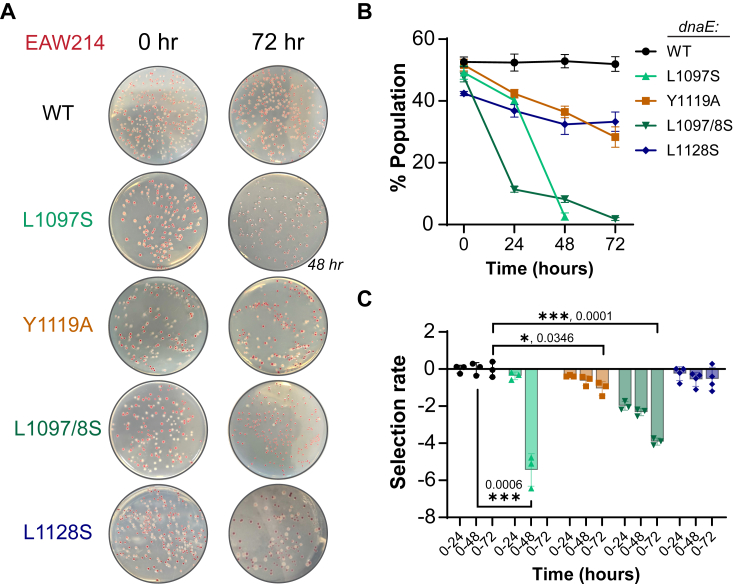
***dnaE*:mut strains demonstrate compromised fitness.***A*, a fitness competition assay in which EAW214 (*red*) and the MG1655 *dnaE* strains (*white*) were cocultured and allowed to compete for nutrients in liquid media. Representative plates are shown for each. *B*, changes in the population of the strains are plotted as a function of time over 72 h for an average of at least three biological replicates of three technical replicates each. SEM is represented by error bars. *C*, the selection rates for each culture were calculated using Equation 3 and plotted. *Black bars* indicate statistically significant differences in *p*-values, indicated and represented by ∗<0.05 and ∗∗∗<0.001 from an unpaired two-sided *t* test.

To investigate whether these doubling time differences also result in weaker fitness of the *dnaE:mut* strains, we cocultured them in combination with an MG1655 parental strain with a deletion in the arabinose isomerase promoter (EAW214:*ΔaraBAD*, *ara*^-^). Colorimetric competition between the strains can be visualized after plating on tetrazolium arabinose (TA) agar plates. The arabinose in these plates reduces the triphenyl tetrazolium chloride (TTC) to red formazan, which becomes a carbon source utilized by EAW214, producing red colony forming units (CFUs). The *dnaE*:muts (*ara*^+^) can use the arabinose reducing sugar directly, leaving the TTC oxidized (colorless) and producing standard white colonies (31). Representative plates are shown for each (Fig. 4*A*). As an initial control, *dnaE*:WT EAW214 (*ara*^-^) and *dnaE*:WT MG1655 (*ara*^+^) strains were cocultured and plated on successive days and showed equal populations over 0 to 72 h, where the MG1655 populations are plotted (Fig. 4*B*). Selection rates for each of the strains were calculated and plotted (Fig. 4*C*). Both *dnaE*:Y1119A *and dnaE*:L1097/8S mutant strains displayed significant negative selection rates at 72 h, at −1.04 and −3.91, respectively, compared to that of WT. Curiously, the *dnaE*:L1097S strain, which displayed similar growth (Fig. 3*C*) and *in vitro* synthesis ability (Fig. 2*D*) had been fully outcompeted by the WT strain the most quickly at only 48 h. Notably, *dnaE*:L1097S would occasionally be outcompeted for growth at either 24 or 72 h but always after the population had reached ≤40%. It is possible that *dnaE*:L1097S would accumulate additional mutations during this extended period of growth that would account for this severe reduction in fitness, unlike most other assays with this strain. It is worth noting that the *dnaE*:L1097S and *dnaE*:L1128S strains were plated 1:1 with EAW214 to have a normal equally distributed starting population at 0 h. However, to achieve an equal 1:1 population of colonies at 0 h for *dnaE*:Y1119A and *dnaE*:L1097/8S, these strains had to be plated 3:1 and 4:1, respectively. This is likely from abundant cell death in these strains, also seen with site-specific mutations in *dnaX* (τ) (30).

### Filamentation of dnaE:mut strains indicate cellular stress

**Figure 5:**
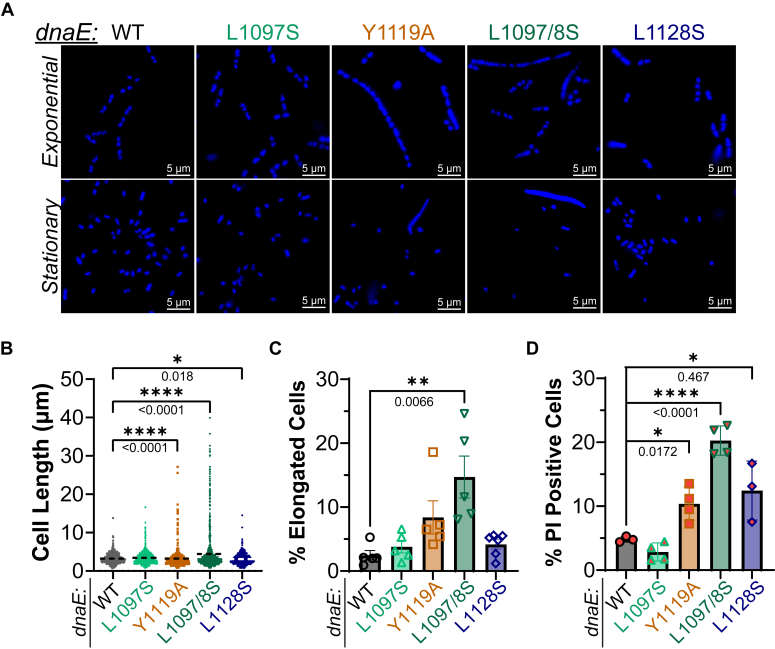
***dnaE*:mut strains display increased cellular elongation and death.***A*, exponential or stationary phase cell populations were stained with DAPI and were imaged using an epifluorescence microscope. *B*, cell lengths were measured for the exponential cell populations (n > 500), and median values were plotted (*dashed black line*). *Black bars* indicate the statistically significant differences in cell lengths calculated from the Kolmogorov-Smirnov test, assuming unequal distributions. The (*C*) % gated elongated and (*D*) PI-positive dead cells from FACS were plotted from at least three biological replicates for 10,000 events each, where the error bars represent the SEM. *Black bars* indicate statistically significant differences calculated from an unpaired two-sided *t* test. *p*-values are indicated and represented by ∗ <0.05, ∗∗<0.01, or ∗∗∗∗ <0.0001. DAPI, 4′,6-diamidino-2-phenylindole; PI, propidium iodide; FACS, fluorescence-activated cell sorting.

To visualize any phenotypic changes to cell morphology, both exponential and stationary phase *dnaE*:mut strains were stained with 4′,6-diamidino-2-phenylindole (DAPI) and imaged with a fluorescence microscope (Fig. 5*A*). Quantification of the cell lengths showed several strains with severe elongation in the exponential phase (Fig. 5*B*). In exponentially growing cells, the MG1655 WT parent strain displayed a median cell length of 3.0 μm. Even though *dnaE*:Y1119A and *dnaE*:L1097/8S strains had median lengths of 2.8 and 3.0 μm, respectively, they also had cells reaching maximum lengths of 27 μm and 40 μm, respectively. Interestingly, the *dnaE*:L1128S strain showed a statistically significant reduction in the median log phase length at 2.7 μm, compared to the parent, even though there were also populations of cells slightly longer than that of WT. The longest cell lengths measured in exponential phase for *dnaE*:WT, *dnaE*:L1097S, and *dnaE*:L1128S were 13.8, 16.7, an 14.5 μm. When gated and analyzed by fluorescence-activated cell sorting (FACS), *dnaE*:L1097/8S had 15 ± 3% of cells that were elongated. All strains showed reduced elongation as cells entered stationary phase (Fig. S7*A*), likely providing the necessary time to repair any genomic damage or cellular stress. Although *dnaE*:L1097S did not have significantly longer cell lengths in exponential phase, the median cell population was significantly longer at 1.5 μm compared to WT at 1.3 μm in stationary phase, suggesting a more moderate form of cellular stress that took longer to alleviate, possibly providing an explanation for why this strain was fully outcompeted in the competition assays after only 48 h (Fig. 4*C*).

To investigate whether this cellular stress also contributed to more cell death in these strains, cells were stained with propidium iodide (PI) and the percentages of membrane-compromised dead cells were quantified. *dnaE*:Y1119A (10 ± 2%), *dnaE*:L1097/8S (20 ± 1%), and *dnaE*:L1128S (11 ± 3%) all showed increases in cell death over the parent (4.8 ± 0.3%) from these exponential populations (Fig. 5*D*). Stationary phase populations showed similar trends with slightly more elevated percentage of dead cells (Fig. S7, *B* and *C*). To determine whether elongated cells correlate more with dead cells, we gated and quantified for both (Fig. S8). Overall, the percentages of dead cells were increased in the elongated populations (Fig. S7*D*), but similar trends between strains still existed. Here, 11 ± 2% of *dnaE*:Y1119A and 20 ± 1% *dnaE*:L1097/8S of singlets are dead compared to 5 ± 1% for WT. For the elongated cell populations, 33 ± 3% of *dnaE*:L1097/8S are dead compared to 11 ± 2% for WT.

### SOS induction is present *in* dnaE:*mut strains*

**Figure 6:**
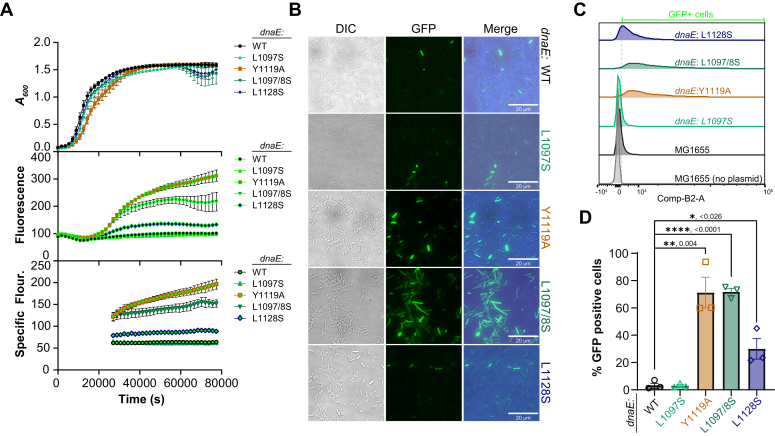
***dnaE*:mut strains display increased SOS.***A*, the growth (*A*_*600*_) and fluorescence (ex 474 nm/em 509 nm) from induction of sgGFP were monitored for *dnaX*:*mut* strains in Miller LB media using a plate reader, allowing the calculation of specific fluorescence (*bottom panel*) using Eq. 4 for an average of 10 wells. Exponential populations of strains expressing sgGFP were examined by (*B*) microscopy or (*C*) FACS (n = 1000 events, each for three biological replicates) and (*D*) quantified for ungated % GFP positive cells with the SEM indicated. *Black bars* show statistically significant differences, where *p*-values are indicated and represented by ∗<0.05, ∗∗<0.01, or ∗∗∗∗<0.0001 from an unpaired two-sided *t* test. FACS, fluorescence-activated cell sorting.

If efficient DNA synthesis by Pol III is disrupted by the *dnaE*:mut strains, then it is likely that significant ssDNA gaps may be present and bound by SSB and RecA to initiate an SOS response to delay cell division (14, 32) consistent with elongated cells seen in Figure 5. To visualize whether *dnaE*:mut strains induce SOS from decoupled DNA synthesis, strains were transformed with a reporter vector, SuperGlo GFP (sgGFP). sgGFP is under the control of a *recN* promoter (one of the 50 or so genes involved in the SOS response) and will induce expression of sgGFP if SOS is activated (33). Strains were grown in 96-wells, and the *A*_*600*_ and fluorescence at 509 nm measured from the plate reader were used to calculate the specific fluorescence according to Equation 4 (Fig. 6*A*) [30]. The *dnaE*:Y1119A and *dnaE*:L1097/8S showed significant increases in specific fluorescence that began during the exponential growth phase and persisted into stationary phase compared to the parental strain. The *dnaE*:L1128S showed a more modest increase in specific sgGFP fluorescence, indicating a minor activation of the SOS pathway, while *dnaE*:L1097S was indistinguishable from the parent.

To better visualize SOS-induced GFP activation, cells were analyzed by fluorescence microscopy (Fig. 6*B*). Both *dnaE*:Y1119A and *dnaE*:L1097/8S appeared to have significantly more GFP positive cells than any of the other strains. Fluorescent microscopy images were quantified blindly for the percent GFP-positive cells (>5000 cells). While *dnaE*:WT had less than 2% positive GFP cells, *dnaE*:Y1119A and *dnaE*:L1097/8S had 21% and 26%, respectively. Briefly, *dnaE*:L1097S and *dnaE*:L1128S had 4% and 11% GFP-positive cells, respectively. To more robustly quantify the GFP-positive cells, we used flow cytometry (Fig. 6*C*). Similar to the plate reader specific fluorescence and microscopy, flow cytometry showed significant increases in GFP-positive cells for Y1119A (71 ± 11%), L1097/8S (71 ± 3%), and L1128S (30 ± 8%) (Fig. 6*D*).

### Increased ssDNA gaps are present *in* dnaE:*mut strains*

**Figure 7:**
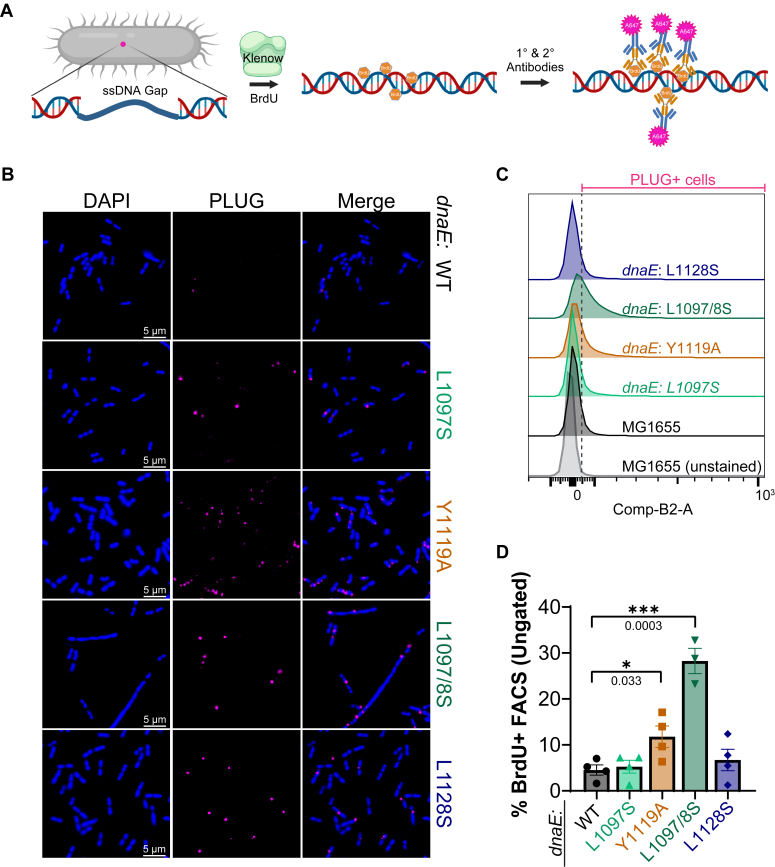
***dnaE*:mut strains show elevated ssDNA gaps.***A*, a schematic of the PLUG technique for detecting ssDNA gaps *in situ* was created on BioRender.com. *B*, log phase strains were stained with DAPI (*blue*) and probed for ssDNA gaps by PLUG (*pink*). PLUG + cells were (*C*) detected by FACS (n = 10,000 events) and (*D*) quantified (for three biological replicates) for the ungated population with the SEM indicated. *Black bars* are used to show statistically significant differences, where *p*-values are indicated and represented by ∗<0.05 or ∗∗∗<0.001 from an unpaired two-sided *t* test. DAPI, 4′,6-diamidino-2-phenylindole; PLUG, PoL I dUTP Gap filling assay; FACS, fluorescence-activated cell sorting.

A PoL I dUTP Gap filling assay (PLUG) assay (Fig. 7*A*) was used to visualize ssDNA gaps of *dnaE*:mut and *dnaE*:WT MG1655 mid-log phase (*A*_*600*_ = ∼0.4) cells (29, 34). ssDNA gaps are filled in with bromodeoxyuridine (BrdU) nucleotides *in situ* using Klenow (exo-) polymerase and then detected with fluorescent antibodies. Fluorescence microscopy is used to show the relative distribution of PLUG foci within the cell populations for each strain (Fig. 7*B*). The *dnaE*:L1097/8S and *dnaE*:Y1119A strains appeared to show the highest amounts of BrdU foci, followed by the *dnaE*:L1128S and *dnaE*:L1097S strains compared to *dnaE*:WT. To confirm these observations, BrdU-labeled cells were quantified by flow cytometry for 10,000 events (Fig. 7*C*). *dnaE*:WT had a mean population of 5 ± 1% cells containing PLUG-detected ssDNA gaps. The *dnaE*:L1097S strain displayed an identical percentage of cells with ssDNA gaps to that of WT, while the *dnaE*:L1128S strain was slightly elevated at 7 ± 2%. Both the *dnaE*:Y1119A and *dnaE*:L1097/8S strains showed significantly greater proportions of PLUG-positive cells at 12 ± 2% and 28 ± 2%, respectively. These PLUG values are less than those reported for SOS-positive cells (Fig. 6*D*) and may be an underrepresentation of the BrdU-positive cells. In fact, the length of exposed ssDNA, the number of ssDNA gaps present, and the length (*i.e.*, filamentation) of cells will all contribute to gating parameters for BrdU-positive cells. Moreover, the PLUG technique relies on the effective incorporation of BrdU nucleotides within fixed cells and the detection by α-BrdU antibodies. We estimate that the current PLUG technique is >60% effective in detecting cells with ssDNA gaps by FACS. However, when compared to the parental strain, it is clear that both *dnaE*:Y1119A and *dnaE*:L1097/8S have significantly more ssDNA gaps created by decoupled DNA Pol III synthesis.

## Discussion

Replisome coupling interactions between the Pol III α-subunit and the CLC τ-subunit are vital for rapid and efficient DNA replication to maintain genome stability; however, the consequences of disrupting this interaction *in vivo* were not known. When the replisome becomes decoupled as Pol III loses its connection with DnaB through τ, stretches of ssDNA are left unreplicated as DnaB continues to unwind. DnaB is at the forefront of the replisome and remains associated with the replication fork for the longest duration (6, 12, 35). It acts as a stable platform around which the rest of the replisomal enzymes assemble to perform their more dynamic duties. τ appears to interact more directly with a constricted form of DnaB for fast unwinding (36), but τ has also been shown to help dynamically regulate the DnaB confirmation to transverse over duplex DNA when needed (30), allowing coupling to be maintained with the polymerase through τ′s flexible linker in domains IV and V (Fig. 1). In fact, complementary mutations in the C-terminus of τ show similar phenotypic cellular stress responses (30) to those shown with mutations of α-Pol III described herein. Therefore, it is important to maintain a physical linkage between the helicase and the polymerase to couple and recouple unwinding with synthesis to respond to physical and kinetic challenges to contiguous replication, thus reducing the production of toxic ssDNA stretches.

DNA replication can be performed at speeds up to 1000 nt s**^−^**^1^, requiring that replisomal proteins optimally synchronize their binding, exchange, and enzymatic activities, creating a highly dynamic replisome (19, 37). Contrary to previous models suggesting that polymerases are more stably associated to the replisome, more recent single molecule studies exposed the stochastic behavior of polymerases that display frequent exchanges of Pol III core enzymes, untethered to the β-sliding clamp when excess Pol III core is in solution (35, 38, 39). The Pol III core exchange rate is concentration dependent (40), and the amount of Pol III core available in solution will determine the rate of exchange implying a complex multiequilibria cellular process. The Pol III core has a very low processivity on its own (41), and without the β-sliding clamp to bind to and promote greater stability on DNA (42), the ability of Pol III core to rapidly synthesize DNA continuously is greatly compromised. Mutations in the C-terminus of α-Pol III not only disrupt the link between the polymerase and τ-CLC but result in a severed connection to the helicase. This disrupted communication implies that a constricted DnaB, still bound by τ-CLC, will continue to unwind at a faster rate, leaving vast regions of ssDNA exposed. Accumulation of ssDNA is a signal for the SOS response and functions to induce gene expression of DNA repair genes that contribute to error-prone DNA synthesis for survival (13, 43). Disruption of the α-Pol III-τ-CLC connection results in abundant ssDNA gaps that induce the SOS response, create cellular filamentation phenotypes, and increase cell death in *E. coli*.

To better understand the cellular and genomic consequences of decoupling, we introduced these α-Pol III mutations chromosomally into the *dnaE* gene. Importantly, the most severe phenotypes were observed with complementary *dnaE* mutants (Y1119A, L1097/8S, and L1128S), where the most decoupling was observed *in vitro*. In particular, *dnaE*:Y1119A and *dnaE*:L1097/8S exhibited the most severe phenotypes, while *dnaE*:L1128S was more moderate. MG1655 easily outcompeted those strains; however, we are unable to explain why the single *dnaE*:L1097S mutant strain was the most rapidly outcompeted after only 48 h. This observation was repeated several times with the same result. It may be that with this extended time frame, *dnaE*:L1097S is either accumulating mutations that are undetected in other shorter assays or that this mutation is affecting other yet unknown pathways for cell survival. One hint may be that *dnaE*:L1097S exhibits a subtle but significant 20% increase in cell length that extends only in stationary phase, which is unlike any of the other strains, where any filamentation is resolved either by repair mechanisms or cell death in stationary phase (Fig. S7*A*).

The most striking phenotypes include significantly elongated cell populations for both *dnaE*:Y1119A and *dnaE*:L1097/8S, which correlate with more cell death. These elongated cellular phenotypes indicate excessive genomic stress induced during decoupled DNA synthesis in the exponential phase. This is witnessed by a striking and strong activation of the SOS pathway in the majority of cells in these affected strains detected in several complementary assays. Details on the type of genomic stress can be gleaned from our PLUG assay, where both strains exhibited increased ssDNA gaps resulting from decoupled unwinding and synthesis generated by these mutations. Relating these genomic consequences back to the structural contacts indicates that mutation of L1097S by itself is not destabilizing enough to cause significant immediate genomic consequences even though it is in the middle of this interaction space (Fig. 1*B*). Rather, more significant surface area contact disruption is required (*i.e.* Y1119A or L1097/8S) to cause decoupling.

The combination of *in vitro* biochemistry and *in vivo* cellular investigations provides an unmitigated approach to probing, defining, and validating important contacts within the replisome. To date, we have probed the role of DnaB helicase regulation (6, 29), mutations in the complementary region of τ domain V (30), and now these opposing mutations in α-Pol III. Hence, we have explored the consequences of decoupling within the *E. coli* replisome from the perspective of three enzymatic angles. Briefly, mutations in DnaB mutants enforced a more constricted conformation of the hexamer, resulting in rapid DNA unwinding, separation from the Pol III holoenzyme, and the production of long stretches of ssDNA accumulation (6, 29). Of the four DnaB mutants studied, K180A and RR329/8AA have the most severe phenotypes and are essentially fully constricted forms of the enzyme that activate faster dysregulated unwinding, creating ssDNA gaps, SOS induction, increased mutational frequencies, altered chromosomal complexities, and cell death.

In the WT situation, the interaction between τ and α is stronger than that for τ and DnaB, and so, the CLC will remain primarily tethered to α-Pol III as a holoenzyme complex and only discontinuously interact with DnaB to regulate its speed of unwinding when DNA priming occurs. Mutations in τ (domain V) would theoretically alter that equilibria in favor of τ (domain IV) binding to DnaB activating a primarily constricted state that can occasionally dilate to overcome obstacles to unwinding, but we also found that τ Domain V mutations also disrupted the ability for DnaB to dilate and translocate over duplex DNA stretches (30). Although τ Domains III and IV interact more strongly with DnaB to impose faster unwinding (44), Domains V may also dynamically regulate the DnaB N-terminal domain collar conformation to control unwinding. In support, τ-domain V (*dnaX*) mutants, S617P and L635P/D636G, displayed the most severe cellular and genomic phenotypes, including extensive cellular filamentation, robust SOS response, increased ssDNA gaps, and cell death (30). Interestingly, the *dnaX* mutations did not display significant increases in chromosome complexities like that for *dnaB* mutants (6), indicating fewer recombinatorial events. In fact in the constricted *dnaB* mutant strains, RecA is needed to mediate ssDNA gaps that are generated and to prevent more severe double-strand breaks from occurring but with a cost of increased mutagenesis stimulated by the SOS response and more genomic recombination (29).

In most cases, mutations in *dnaE* (α-Pol III) appear to be similar to the complementary mutations in *dnaX*; however, α-Pol III mutations are far worse in leading strand TFII rolling circle assays. It may be that these α-Pol III mutations are losing the coupling connection with DnaB because the WT τ-CLC in these assays enforces faster unwinding by DnaB. Conversely, mutations in τ-CLC disrupt interactions with Pol III and DnaB, but this does not negatively affect leading strand synthesis. In fact, τ-CLC (L635P/D636G) routinely exhibited slightly faster and longer leading strand products, possibly indicating that τ is needed to regulate replisome speed for primase recruitment and lagging strand synthesis (45). How the genomic stress is alleviated when the replisome decouples stochastically likely takes contributions from multiple DNA repair/restart pathways dependent on the type of situation created. Future directions will seek to answer how genomic stress resulting from multifaceted replisome decoupling is acknowledged and countered by various repair/restart pathways in bacteria.

## Experimental procedures

### Cloning, expression, and purification of the Pol III **α**-subunit and core mutants

Each of the four Pol III α-mutants was quickchanged from the pET16b-*dnaE*(α)-^HIS^*holE*(θ)-*dnaQ*(ε) or pCDFduet-^HIS^*dnaE*(α)-*holE*(θ) (Table S1) plasmids using partially overlapping forward and reverse primers containing a unique restriction site for screening (Table S2) with Platinum SuperFi II DNA Polymerase (Thermo Fisher Scientific) ligation free protocol (46). Protein purifications of the Pol III core complexes or Pol III α-subunit only were carried out similarly (15). Briefly, plasmids were transformed into either Acella2 (Pol III core) or Rosetta Blue DE3 (α-Pol III) expression cell lines, grown in 2xYT media at 37 °C and 220 rpm, and induced with IPTG (0.5 mM) at *A*_*600*_ = 0.7 for 4 h (Pol III core) or overnight (α-Pol III). Cells were pelleted at 4000*g* for 12 min at 4 °C and stored at −80 °C. Cell pellets are resuspended in 5 ml of His A buffer [20 mM Hepes, 500 mM NaCl, 50 mM imidazole, 10% glycerol, pH 7.5] per gram of pellet, lysed by French press, and incubated with benzonase at 4 °C. Clarification of the lysate is carried out by high-speed centrifugation at 22,000 rpm for 30 min at 4 °C. Pellets are discarded, and the supernatant is filtered through a 0.45 μm aqueous filter before loading onto a 5-mL His-Trap HP affinity column (Cytiva). The column is washed with His B Buffer [20 mM Hepes, 50 mM NaCl, 50 mM imidazole, 10% glycerol, pH 7.5] and Pol III is eluted either by ramping to 100% His C buffer [20 mM Hepes, 50 mM NaCl, 500 mM imidazole, 10% glycerol, pH 7.5], where the peak is collated all in one fraction tube (Pol III core) or eluted using a step-wise gradient (α-Pol III). The elution is loaded onto a 1-mL Hi-Trap Heparin HP column (Cytiva) equilibrated with heparin A buffer [50 mM Hepes, 0.1 mM EDTA, 5 mM β-mercaptoethanol, 10% glycerol, pH 7.5]. Pol III is eluted at 50% heparin B buffer [heparin A buffer, 1 M NaCl] in one fraction and loaded onto a Hi Load Superdex 200 16/600 size exclusion column (Cytiva) in Storage buffer [30 mM Hepes, 100 mM NaCl, 0.5 mM EDTA, 5 mM β-mercaptoethanol, and 20% glycerol, pH 7.5]. Fractions containing Pol III are identified in a 10% SDS-PAGE gel, pooled, and concentrated. The concentrations were determined using *A*_260_ values (ε = 99,920 M^−1^ cm^−1^) from a Nanodrop 2000c (Thermo Fisher Scientific), and the *A*_260_/*A*_280_ ratios were all determined to be below 0.8 (indicating little contaminating DNA) before being flash frozen in liquid nitrogen and stored at −80 °C.

### Expression and purification of **τ**-CLC

The pCOLADuet−1 plasmid (Table S1) containing the *dnaX* gene (47) was transformed into Rosetta2 expression cells, and overexpression of τ_3_δδ’ψχ was induced with IPTG (1 mM). Purification was performed as previously described (30, 47). The concentrations were determined using *A*_260_ values (ε = 302,150 M^−1^ cm^−1^), and the *A*_260_/*A*_280_ ratios were all determined to be below 0.8 before being flash-frozen in liquid nitrogen and stored at −80 °C.

### Nickel pull-down assays for **α-τ**

Pull-down assays were performed by adding 50 μl of resuspended nickel resin (HisPur Ni-NTA Resin, Thermo Fisher Scientific) to a 1.5 ml Eppendorf tube and centrifuged at 700*g* for 2 min. The supernatant is removed, the pellet is resuspended in 50 μl of binding buffer [20 mM sodium phosphate, 0.5 M NaCl, 20 mM imidazole, pH 7.4], centrifuged, and the supernatant discarded. Then, a 1:2 M ratio of τ-CLC and Pol III-α was mixed with the resin and allowed to rock at 4 °C for 30 min before centrifuging and transferring the flow through solution to a new tube. Next, the resin pellet is resuspended in 50 μl of binding buffer, centrifuged, and transferred to a new tube as the first wash, taking care not to disturb the resin pellet. This wash step is repeated 12 more times to ensure that all unbound protein is removed before resuspending the resin in 50 μl of elution buffer [20 mM sodium phosphate, 0.5 M NaCl, 500 mM imidazole, pH 7.4], centrifuging, and transferring the elution solution to a new tube. Finally, the flow through, washes, and elution are run on a 10% SDS-page gel, and the proteins are detected by a Western blot with primary antibodies to α or τ (24) and the secondary antibody, Cy5 goat anti-rabbit (ab6564, Abcam), and visualized on the Typhoon RGB gel imaging scanner (Cytiva).

### Pol III core primer extension experiments

Primer extension assays were performed as described (48) with DNA52 as the template and DNA22 as the primer (Table S2) (Integrated DNA Technologies). Primer template annealing is performed by combining the oligos in a 1:1.2 primer: template ratio, heating to 95 °C, and allowing to cool to room temperature overnight. All polymerization reactions are conducted in Pol III core reaction buffer [50 mM Hepes, 80 mM NaCl, 2 mM DTT, 0.1 mM EDTA, 0.1 mg/ml bovine serum albumin, pH 7.4]. For each reaction, 200 nM Pol III core is loaded onto the substrate in 10 μl volumes, incubated at 37 °C for 5 min, initiated with or without 200 μM dNTPs and 10 mM MgOAc, allowed to react for the allotted time before quenching with 500 mM EDTA. Extended or degraded DNA products are separated on a large DNA sequencing gel [1X tris-borate-EDTA buffer, 20% acrylamide, and 7 M urea] and run at a constant 40 W for 3 h. Cy5 signals were visualized on a Typhoon RGB gel imaging scanner (Cytiva). Extension and degradation products were quantified by ImageQuant (Cytiva, v10.1) and plotted using GraphPad Prism (v10.3), where the error bars represent the SEM.

### *In vitro* whole replisome TFII rolling circle synthesis assay

Leading strand rolling circle assays were carried out by reconstituting the *E. coli* replisome on a TFII substrate prepared from the vector pSCW01 (Table S1) as described (28). To make the gapped plasmid, 100 μg of pSCW01 plasmid, 22.5 units of Nt.*Bst*NBI, 1X NEB 3.1 buffer, and 40 μM of each of the following oligomers: DNA197, DNA198, and DNA199 (Table S2) were combined in a PCR tube, placed in a thermal cycler at 55 °C for 4 h, 85 °C for 10 min and then reduced to 12 °C (−1 °C/min). A diagnostic restriction digest was performed with BamHI, PstI, and NcoI to validate the gapped plasmid (Fig. S3). The remaining reaction is mixed with an equal volume of 2X PEG solution [26% w/v PEG 8000 and 20 mM MgCl_2_], centrifuged for 1 h at 21,000 g, 6 °C; the supernatant was discarded, and the pellet washed with 1.5 ml of 70% (v/v) ice-cold ethanol. The solution was centrifuged for 30 min at 21,000*g*, 6 °C, and the supernatant was discarded. The pellet was allowed to air dry until no residual ethanol remained. The pellet was resuspended in 80 μl of 65° C sterile water until it fully dissolved. In addition, 80 μl of gapped plasmid, 10X NEB CutSmart buffer, and 2 μM Cy5-DNA200 (Table S2) (Integrated DNA Technologies) were mixed to a final volume of 100 μl and incubated at 50 °C for 10 min, temp decreased by 1 °C/min, and then held at 16 °C. Subsequently, 8 mM ATP, 2.4 ml of T4 DNA ligase (New England Biolabs), and 10 mM DTT were added and incubated at 16 °C for 18 h. The PEG and ethanol precipitation steps were repeated before resuspending the pellet in 100 μl of 1X Tris-EDTA buffer and storing at −20 °C.

*E. coli* DnaB and DnaC were purified as described (6, 49). SSB, beta clamp, CLC (τ_3_δδ’ψχ) were also purified as described (15, 30). For the leading strand rolling circle assay, 4 nM DNA template was incubated with 30 nM CLC (τ_3_δδ’ψχ), 90 nM Pol III core (αεθ), 60 nM DnaB, 360 nM DnaC, and 200 nM beta clamp (β) at 37 °C for 5 min in replication buffer [30 mM Tris–HCl pH 7.6, 12 mM Mg(OAc)_2_, 50 mM potassium glutamate, 0.5 mM EDTA, 0.025% (v/v) Tween-20, and 10 mM DTT] and initiated with 1 mM ATP, 50 nM SSB, and 125 μM dNTPs. The reactions were quenched with equal volumes of 2x DNA gel loading dye, 200 mM EDTA (LES buffer) at various time points. Quenched samples were electrophoresed on a 0.5% alkaline agarose gel 15V for 16 h. Gels were neutralized by rocking at RT in 2xTris-acetate-EDTA buffer for 2 h and stained with 0.2X SYBR Gold (Invitrogen) at RT for 2 h. Gels were imaged using a Typhoon RGB gel imaging scanner (Cytiva) and quantified by ImageQuant (Cytiva, v10.1).

### Bacterial CRISPR-Cas9 genome editing

The parental *E. coli* strain, MG1655, was used to create genetically edited CRISPR/Cas9 mutants of the *dnaE* gene using a dual vector targeting system (strains listed in Table S3). Synthetic ∼40 base oligonucleotides with sequences homologous to the *dnaE* target region acting as guide RNA (Table S2) were ligated into the pCRISPR plasmid (Addgene: 42875) (50). Subsequently, 100 ng of pCRISPR-g*dnaE* and 1 μM of the editing oligonucleotide (Table S2) containing the homologous region to *dnaE*, targeted single point mutations, and novel restriction sites for screening were simultaneously electroporated into the MG1655 parent strain containing pREDCas9 (Addgene: 71541) (51), and plated on spectinomycin/kanamycin (50 μg/ml) LB agar plates (10 g/L tryptone, 5 g/L yeast extract, 10 g/L NaCl, and 15 g/L agar) and incubated at 30 °C. A colony PCR and restriction enzyme digest were used to verify the incorporation of the editing oligo into the genome by cas9, thus confirming mutagenesis (Fig. S5). DNA sequencing of these PCR amplicons further confirmed successful editing (Plasmidsaurus). These *dnaE*:muts strains were then cured of the pCRISPR and pREDcas9 vectors, respectively, by first antibiotic streaking on spectinomycin and kanamycin LB plates and then streaking on spectinomycin *versus* LB plates at 42 °C as pREDCas9 contains a temperature sensitive origin, p15A. All other experiments were performed at 37 °C unless otherwise indicated.

### Growth assays

Overnight growth cultures were diluted 1 to 200 μl in either M9 minimal media [20 ml of 5X M9 salts, 2 ml of 20% glucose (sterile filtered), 200 μl of 1 M MgSO_4_, 10 μl of 1 M CaCl_2_] or LB media [10 g/L tryptone (RPI, molecular biology grade), 5 g/L yeast extract, 10 g/L NaCl]. Growth assays were performed using white, clear, and flat-bottomed 96-well plates and analyzed on a Tecan Spark microplate reader. The *A*_*600*_ absorbance values were recorded at 30-min intervals over a course of 24 h at 37 °C, 240 rpm to control for regular growth profiles, and a humidity cassette to prevent changes in media volume. Data were analyzed using GraphPad Prism (v10.3) and fit into a modified 4-parameter Gompertz growth model as follows:

(1) $$w\left(t\right)=B+A^{{-e}^{\left(\frac{k_{g}\times 2.7182}{A}\times \left(T_{lag}-t\right)+1\right)}}$$

where *w(t)* is the density as a function of time, *B* is the lower asymptote, *A* is the higher asymptote, *T*_*lag*_ is the lag time of the culture, *t* is time, and *k*_*g*_ is the growth rate coefficient. The specific growth (μ) is calculated from a rolling average over 30 min. The absolute growth rate (*k*_*z*_) can be calculated using the following equation:

(2) $$k_{z}=\frac{k_{g}\times 2.7182}{A}$$

and is equivalent to *μ*_*max*._ The absolute growth rates were plotted and analyzed for any significant differences using an unpaired two-tailed *t* test with GraphPad Prism (v10.3).

### Competition and fitness assay

An MG1655 derivative, EAW214 (Table S3), containing an *araBAD* mutation within the promoter region, was used as the control strain for this assay (31). The *dnaE*:WT (MG1655) and EAW214 strains were mixed in equal parts based on their absorbance from an overnight culture. Mixed populations were diluted 10^-6^ in ultrapure sterile water and plated on TA plates contain 1% arabinose (Oakwood products) and 0.2 mg/ml TTC (Sigma-Aldrich)], to ensure a 50% population of MG1655 and EAW214 colonies, within 10%, on day 0. The overnight cultures were diluted 100-fold in LB media, mixed in equal parts, allowed to grow for 24 h, diluted, and plated. This procedure was repeated for 72 h or until one population had been fully outcompeted (6, 31). The *dnaE*:Y1119A and *dnaE*:L1097/8S *strains* were mixed in 3:1 or 4:1 ratios, respectively, with EAW214, to achieve 50% populations on day 0. The selection rate of each of the strains was calculated using the following equation:

(3) $$selection\;rate=r=\ln\frac{A_{1}}{A_{0}}-\ln\frac{B_{1}}{B_{0}\;}$$

where *A*_*0*_ and *B*_*0*_ are CFU fractions of strains A and B at time 0, and *A*_*1*_ and *B*_*1*_ are CFU fractions of strains A and B at 24, 48, and 72 h. The selection rates were plotted and analyzed for significance using an unpaired two-tailed *t* test with GraphPad Prism (v10.3).

### Live/dead analysis

To examine the viability of strains, stationary phase cells were stained with PI to measure the percentage of dead cells in the population. Cultures were grown overnight, and 1 ml of culture was withdrawn, pelleted, and washed 3 times with sterile PBS. The pellet was resuspended in PBS, stained with 3 μM PI, and incubated in the dark for 15 min at room temperature. The unstained negative control, *dnaE*:WT, was pelleted and washed 3 times with sterile PBS, resuspended in PBS, and was not treated with PI. The fixed/stained positive control was processed the same as the negative control; however, after pelleting the cells and washing with PBS, the cells were resuspended in 70% ethanol to permeabilize and fix the cells before staining with 3 μM PI. Samples were diluted with sheath fluid and analyzed by flow cytometry (Northern Lights flow cytometer, Cytek). Unstained cells were used for gating. Data were plotted using FlowJo 10.6.1 (BD Biosciences).

### Cell morphology and length measurements

Stationary phase cells from overnight grown cultures or exponential cells grown from diluted overnight cultures were pelleted, washed three times with 1x PBS, and resuspended in 1 ml of 70% ethanol. Subsequently, 3 μl of the bacterial suspension was spotted on a microscope slide, left to air dry, and 2 μl of SlowFade Diamond Antifade Mountant with DAPI (Thermo Fisher Scientific) was added and then covered immediately with a coverslip and sealed with clear nail polish. Microscopy images were visualized using an Olympus Brightfield Microscope IX-81 (Olympus Corp) with a 60x objective lens upon oil immersion. Cell filamentation lengths were quantified using ImageJ (v1.53) (https://imagej.net/ij/) using a blinded approach. The scale was set using the included scale bar from each microscopy image. Lengths were measured using the segmented line tool to account for cell curvature, with the length being the longest diameter of a given cell. The cell lengths were plotted and analyzed for statistical differences in the distributions using the Kolmogorov-Smirnov test with GraphPad Prism (v10.3).

### SOS experiments

The *dnaE*:mut strains were transformed with plasmid pEAW915 (gift from Mike Cox), containing the SuperGlo GFP gene under the SOS-controlled *recN* promoter (33). Subsequently, 2 μl of overnight-grown cultures was added to 200 μl aliquots of LB media in white, clear-bottomed 96-well plates. The cultures were incubated at 37 °C, and the absorbance (*A*_600_) and fluorescence (ex 474 nm/em 509 nm) were read at 30-min intervals over 24 h using a Tecan Spark microplate reader. The specific fluorescence was calculated using the following equation.

$$Specific\;fluorescence=\frac{Fluorescence}{Absorbance\;\left(A_{600}\right)}$$

To analyze SuperGlo GFP fluorescence cells directly by microscopy, 200 μl of overnight grown cultures were pelleted and washed three times with 1x PBS, fixed with 4% paraformaldehyde for 20 min, and incubated at room temperature. The fixed cells were pelleted, washed thrice with 1x PBS, and resuspended in 300 μl of 1x PBS. Briefly, 3 μl of the bacterial suspension was spotted on a microscope slide, and the slides were prepared and visualized as above.

To quantify the SuperGlo GFP-expressing cells globally within the population, overnight cultures were pelleted, washed three times with 1x PBS, resuspended in 1 ml of 1x PBS, and analyzed using a flow cytometer (Cytek Northern Lights flow cytometer). Data were plotted using FlowJo10.6.1 (BD Biosciences).

### PLUG experiments

The PLUG immunofluorescence assay was performed as described (34), using a BD FACSVerse flow cytometer (BD Biosciences) and analyzed by FlowJo v10.6.1 software. Microscopy was performed by spotting slides with 3 μl of cell sample followed by 2 μl of staining and the mounting solution containing DAPI (Thermo Fisher Scientific), covering with a round coverslip, and sealing with clear polish. Slides were placed in the dark at 4 °C and allowed to set overnight for better images. An Olympus Brightfield Microscope IX-81 (Olympus Corp), with an oil immersion 60x objective lens, was used to visualize images.

## Data availability

All biochemical data presented in this study, including values, gels, images, datasets, and any strains or plasmids, are available upon request to the corresponding author.

## Supporting information

This article contains supporting information (6, 15, 28, 33, 47, 52, 53, 54, 55, 56).

## Conflicts of interest

The authors declare that they have no conflicts of interest with the contents of this article.
