# Supplementary material for: Replisomal coupling between the α-pol III core and the τ-subunit of the clamp loader complex (CLC) are essential for genomic integrity in Escherichia coli
Source: J Biol Chem. 2025 Jan 10;301(2):108177. doi: 10.1016/j.jbc.2025.108177 (PMC11869525; doi:10.1016/j.jbc.2025.108177)
Supplement: Supporting information [file mmc1.pdf]

SUPPORTING FIGURES AND TABLES

Replisomal coupling between the α-Pol III core and the τ-subunit of the clamp loader complex (CLC) are essential for genomic integrity in *E. coli*

Lauren, J. Butterworth[1], Malisha U. Welikala[1], Cody W. Klatt[1], Kaitlyn E. Rheney[1] and Michael A. Trakselis[1*]

[1]Department of Chemistry and Biochemistry, Baylor University, Waco, Texas, 76798-7348, USA
*To whom correspondence should be addressed: *Michael A. Trakselis, One Bear Place #97348, Waco, TX 76798-7348. Tel 254-710-2581; Email: michael_trakselis@baylor.edu

Table S1: Plasmids

Plasmids	Source	Description
pCDF duet ^{HIS}*dnaE_holE*	(1)	Overexpression of Pol III alpha and theta (αθ)
pET16b_*dnaE*(α)_^{HIS}*holE*(θ)-dnaQ(ε)	(2)	Overexpression of Pol III core (αεθ)
pCOLADuet-1-*dnaX*	(3)	Overexpression of τ_3-CLC used in pull-down experiments
pTC.1.2	(4)	Overexpression of τ_3-CLC used in TFII experiments
pSJS9	(5)	Overexpression of ß-clamp
pET11b DnaB	(6)	Overexpression of DnaB
pET28b-DnaC	(7)	Overexpression of DnaC
pET3-SSB	(8)	Overexpression of SSB
pSCW01	(9)	TFII substrate
pEAW915	(10)	SuperGLo GFP plasmid with the *recN* promoter

Table S2: Oligonucleotides

Name	Sequence (5'- 3')
SDM L1097S Fw	GATGACCAAAGCTTAAACCGACTCCGTCAG
SDM L1097S Rv	GTCGGTTTAAGCTTTGGTCATCATCAATTTG
SDM Y1119A Fw	CATCTCTACGCTCAGCGGGCGGATGCACGC
SDM Y1119A Rv	CGCCCGCTGAGCGTAGAGATGTACTGGAATTG
SDM L1097/8S Fw	GACCAGAGCTCAAACCGTCTACGTCAGTCTC
SDM L1097/8S Rv	GACGGTTTGAGCTTTGGTCATCATCAATTTG
SDM L1128S Fw	GCGCGGTCTCGTTTTGGCGCGAC
SDM L1128S Rv	CAAAACGAGACCGCGCGCGTGCATC
pCDFduet HisTag insert	ATGGGATCCCATCACCATCATCACCATCACCATCATAGTTCTGAACCACGTTTCG
pCDFduet Rv	GGTATATCTCCTTATTAAAGTTAAACAAAATTATTTCTACAGGGGAATTGTTATCCGCTC
DNA52	CGTCCAACATGAAGCTTGCACTCGATCGCTGTATCGCTCGCGCTCGCCTCGC
DNA22	Cy5-GCGAGGCGAGCGCGAGCGATAC
DNA197	ATTTGACTCC
DNA198	CATGGACTCGCTGCAG
DNA199	GAATGACTCGG
DNA200	Cy5-AAAAAAAAAAAAAAAAGAGTACTGTACGATCTAGCATCA
CRISPR L1097S Fw	AAACTCCAGAGACTGACGGAGTCGGTTTAAAAGCG
CRISPR L1097S Rv	AAAACGCTTTTAAACCGACTCCGTCAGTCTCTGGA
L1097S editing oligo	GCTGACGGACAGGCAAATTGATGACCAAAGCTTAAACCGACTCCGTCAGTCTCTGGAAC
CRISPR Y1119A Fw	AAACTGCATCCGCCCTCTGATAGTAGAGATGTACG
CRISPR Y1119A Rv	AAAACGTACATCTCTACTATCAGAGGGCGGATGCA
Y1119A editing oligo	CTCTGGGACAATTCCAGTACATCTCTACGCTCAGCGGGCGGATGCACGCGCGCGGTTGC
CRISPR L1097/8S Fw	AAACTCCAGAGACTGACGGAGTCGGTTTAAAAGCG
CRISPR L1097/8S Rv	AAAACGCTTTTAAACCGACTCCGTCAGTCTCTGGA
L1097/8S editing oligo	CTGACGGACAGGCAAATTGATGACCAGAGCTCAAACCGTCAGTCTCTGGAACC
CRISPR L1128S Fw	AAACGAGGGCGGATGCACGCGGGTTGCGTTTG
CRISPR L1128S Rv	AAAACAAACGCAACCGCGCGCGTGCATCCGCCCTC
L1128S editing oligo	CAGAGGGCGGATGCACGCGCGCGGTCTCGTTTTGGCGCGACGTGGCGTGTCTCTCCGAG
pCRISPR rev	CCGGCCACAGTCGATGAATCCAGAAAAGCGGCC
EcodnaE genome Fw	GCTGGAAAAAGACCGCATACTTATCG
EcoaccA genome Rv	CCGAGATCGGCGAAGATTTTACG

Table S3: Strains

Strains	Genotypes	Description
MG1655	*E. coli* K-12	Wild type parental strain, CGSC# 7740
LJB1	MG1655: *dnaE*:L1097S	Contains a *dnaE* single point mutation
LJB2	MG1655: *dnaE*:Y1119A	Contains a *dnaE* single point mutation
LJB3	MG1655: *dnaE*:L1097/8S	Contains a *dnaE* double point mutation
LJB4	MG1655: *dnaE*:L1128S	Contains a *dnaE* single point mutation
EAW214	MG1655 *ΔaraBAD*	Grows red on arabinose plates

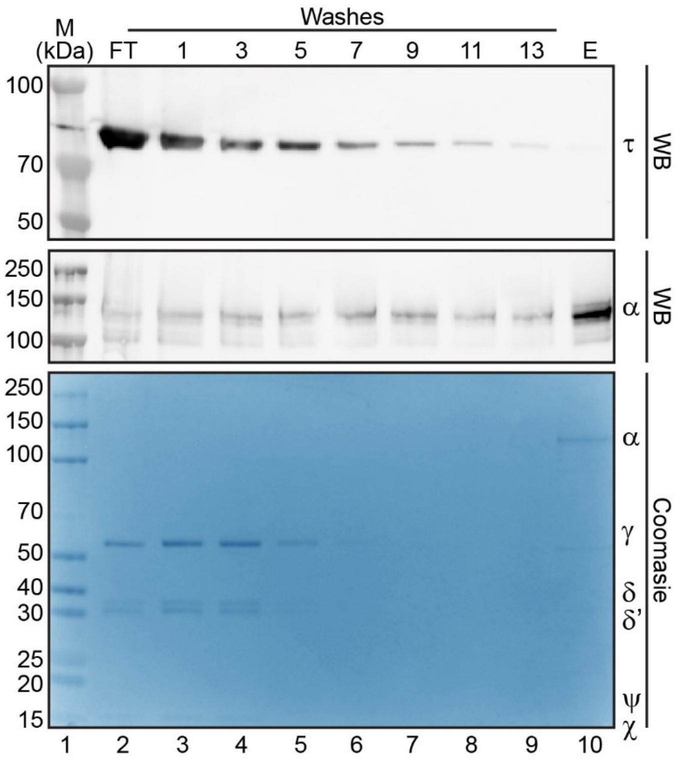

Supporting Figure S1: Nickel pull-down controls. Nickel pull-down experiments of excess His tagged α-Pol III and τ-CLC alone were performed, showing the flow-through (FT), several washes at 20 mM imidazole, and the elution (E). τ alone washes out completely, and α-Pol III alone binds the resin, as detected by western blot (top two gels). Additionally, a nickel pull-down between α-Pol III WT and γ_3-CLC (gift from Charles McHenry) was also performed as a negative control (Coomassie gel).

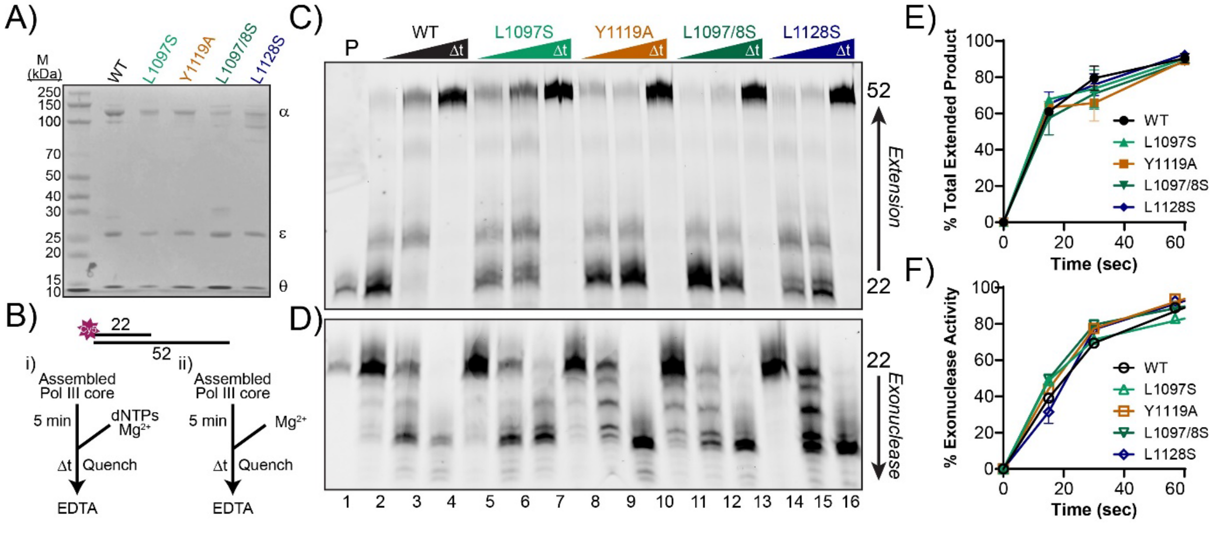

Supporting Figure S2: Normalized DNA Polymerization activity of Pol III core enzyme variants by primer extension assays. To confirm equal activity among Pol III core variants, the DNA synthesis of each (WT, L1097S, Y1119A, L1097/8S, and L1128S) was determined by primer extension ability. The A) SDS page gel shows representative purified Pol III core mutants. B) The substrate utilized a Cy5 labeled 22mer primer annealed to a 52mer template (**Table S3**) and reaction schemes of extension and exonuclease assays. The products for the accompanying C) extension and D) degradation were separated on DNA sequencing gels, E-F) quantified using ImageQuant (v10.1) from triplicate experiments, and plotted using GraphPad Prism where the error bars represent the SEM. There was no significant difference in the polymerization or degradation activity across all variants.

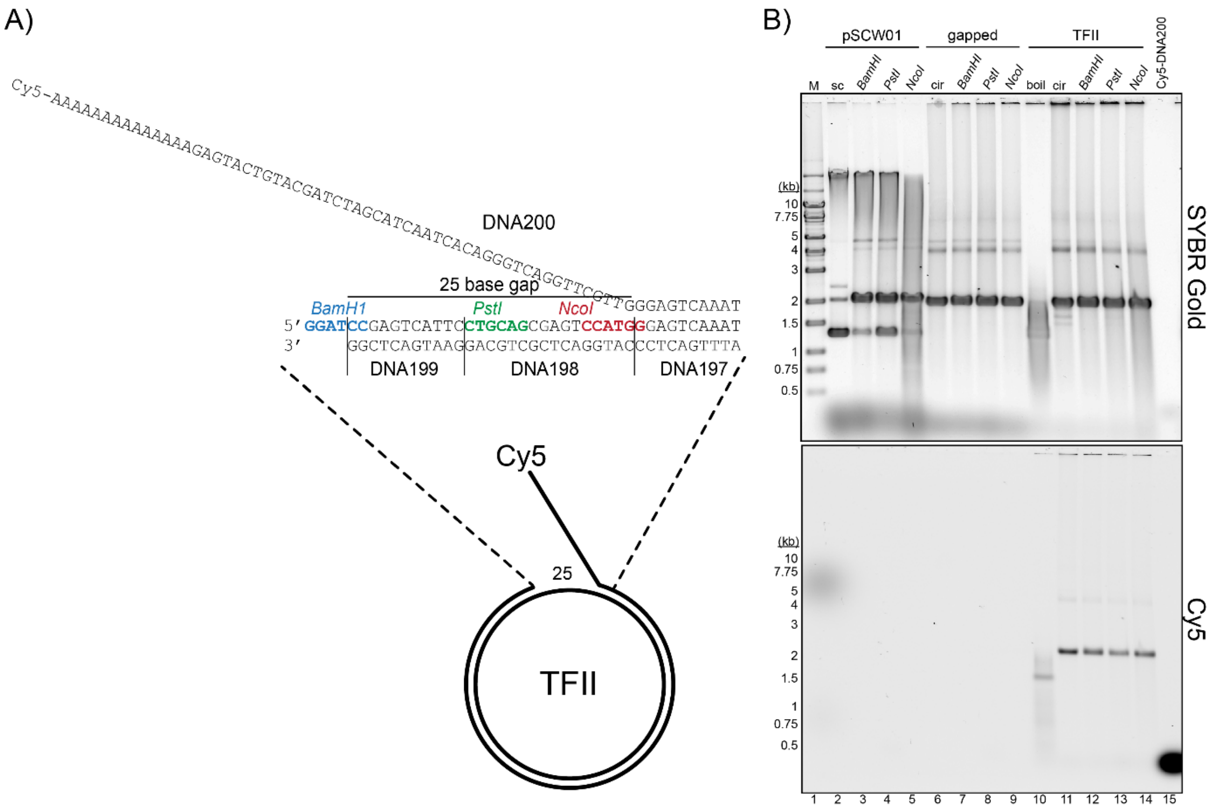

Supporting Figure S3: Tailed Form II (TFII) rolling circle substrate design and analysis. A) The TFII substrate was created using 3 displacer oligos, DNA197, DNA198, and DNA199 (**Table S2**), to create a gapped region on pSCW01. Then a Cy5 labeled flap, DNA200, is annealed and ligated. The presence or absence of the gap is determined by restriction enzyme (RE) digest with 3 enzymes, *BamHI* (blue), *PstII* (green), and *NcoI* (red). B) A 1% agarose 2xTAE gel is electrophoresed to verify the successful creation of the substrate through RE analysis (*lanes 7-9*) and appropriate sizes showing the top (SYBR gold) and bottom (Cy5) image of the same gel. Circular (cir) versions of TFII (*lanes 3-5*), the gapped plasmid (*lane 6*), and supercoiled (sc) pSCW0l plasmid (*lane 2*) are also run for comparison. Finally, a TFII boil (*lane 10*) determines if the DNA200 oligo has been successfully ligated.

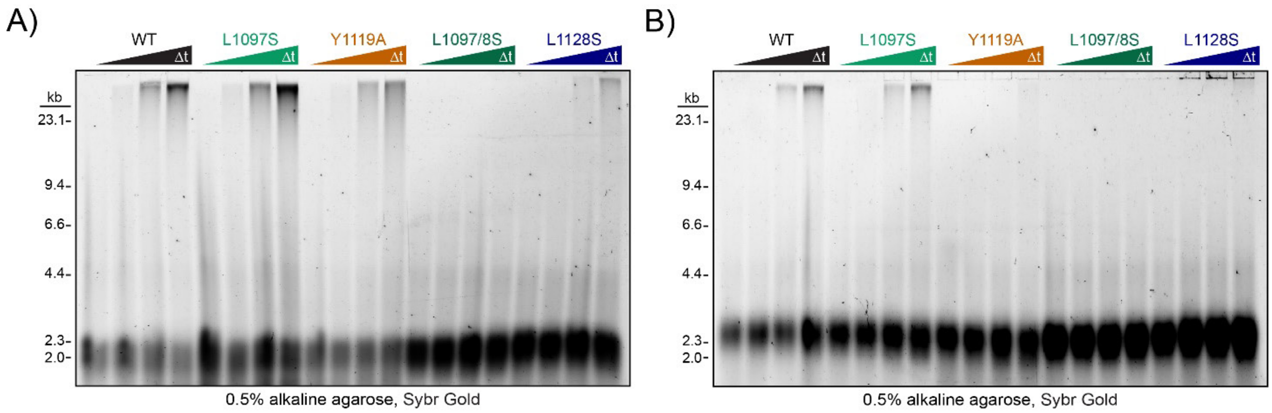

Supporting Figure S4: TFII rolling circle leading strand synthesis replicate gels. The *E. coli in vitro* assembled replisome leading strand replication experiments were performed in triplicate, where the first replicate is shown in **Figure 2** and replicate two A) and three B) agarose gels are shown here.

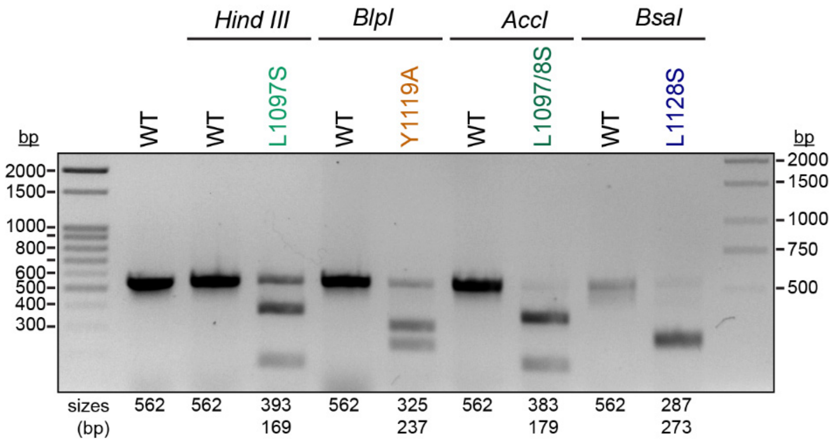

Supporting Figure S5: **Confirmation of *dnaE* genomic editing by restriction digest.** A *dnaE* chromosomal region (~549 bp) was amplified by colony PCR using *EcodnaE* Fw and *EcoaccA* Rv primers (**Table S2**). The *accA* gene (a component of acetyl coenzyme A carboxylase -ACC complex) is downstream from the *dnaE* gene on the *E. coli* chromosome. The amplified *dnaE*:WT and *dnaE*:mut regions were digested by respective restriction enzymes as indicated. Expected band sizes of positive mutagenesis results for each site mutation are 169bp and 380bp (*dnaE*:L1097S), 237bp and 312bp (*dnaE*:Y1119A), 179bp and 370bp (*dnaE*:L1097/8S), and 274bp and 273bp (*dnaE*:L1128S). The target colony PCR amplicons for each were confirmed by sequencing.

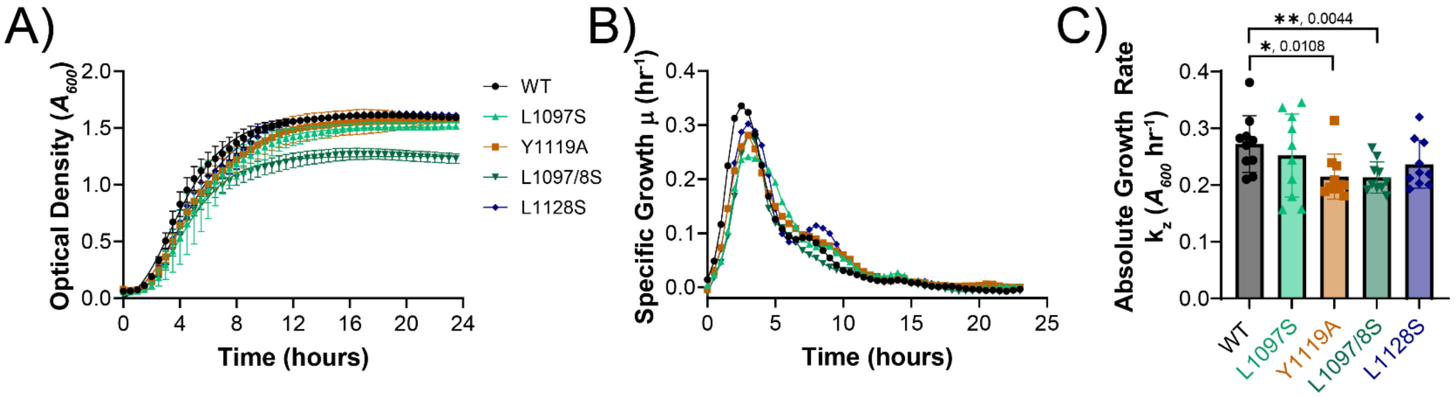

Supporting Figure S6: *dnaE* strains 24-hour growth profiles in LB media. A) The mass doubling of each strain was monitored by A_{600} in 96-well plates. B) The specific growth (μ) was determined from the derivative of the curve over a rolling 30-minute time. C) The maximal growth rate (k_g) was determined from Equation 1 and plotted for the ten technical replicates. Error bars represent SD; the black bars indicate statistically significant differences with *P*-values indicated and represented by *<0.05 and ****<0.0001 from an unpaired two-sided *t*-test.

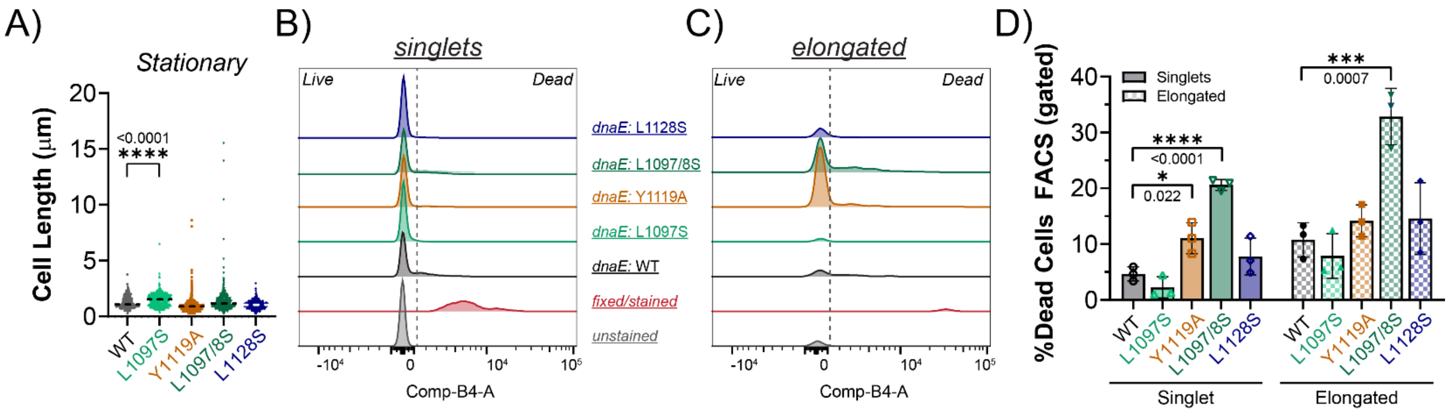

Supporting Figure S7. *dnaE*:mut strains display increased cellular filamentation and death in stationary phase. A) Stationary phase cell populations were stained with DAPI and were imaged using an epifluorescence microscope. Cell lengths were measured for both populations (n > 500), and median values were plotted. Black bars indicate the statistically significant differences calculated from the Kolmogorov-Smirnov test, assuming unequal distributions. The histograms of B) singlet and C) elongated gated live and dead (PI-positive) populations from stationary phase populations by FACS for 10,000 events are shown. D) The percent dead cells from either the singlet or elongated gated stationary phase populations are quantified from three separate biological replicates (for 10,000 events each). Black bars indicate statistically significant differences calculated from an unpaired two-sided *t*-test. *P*-values are indicated and represented by * <0.05, ***<0.001, or **** <0.0001.

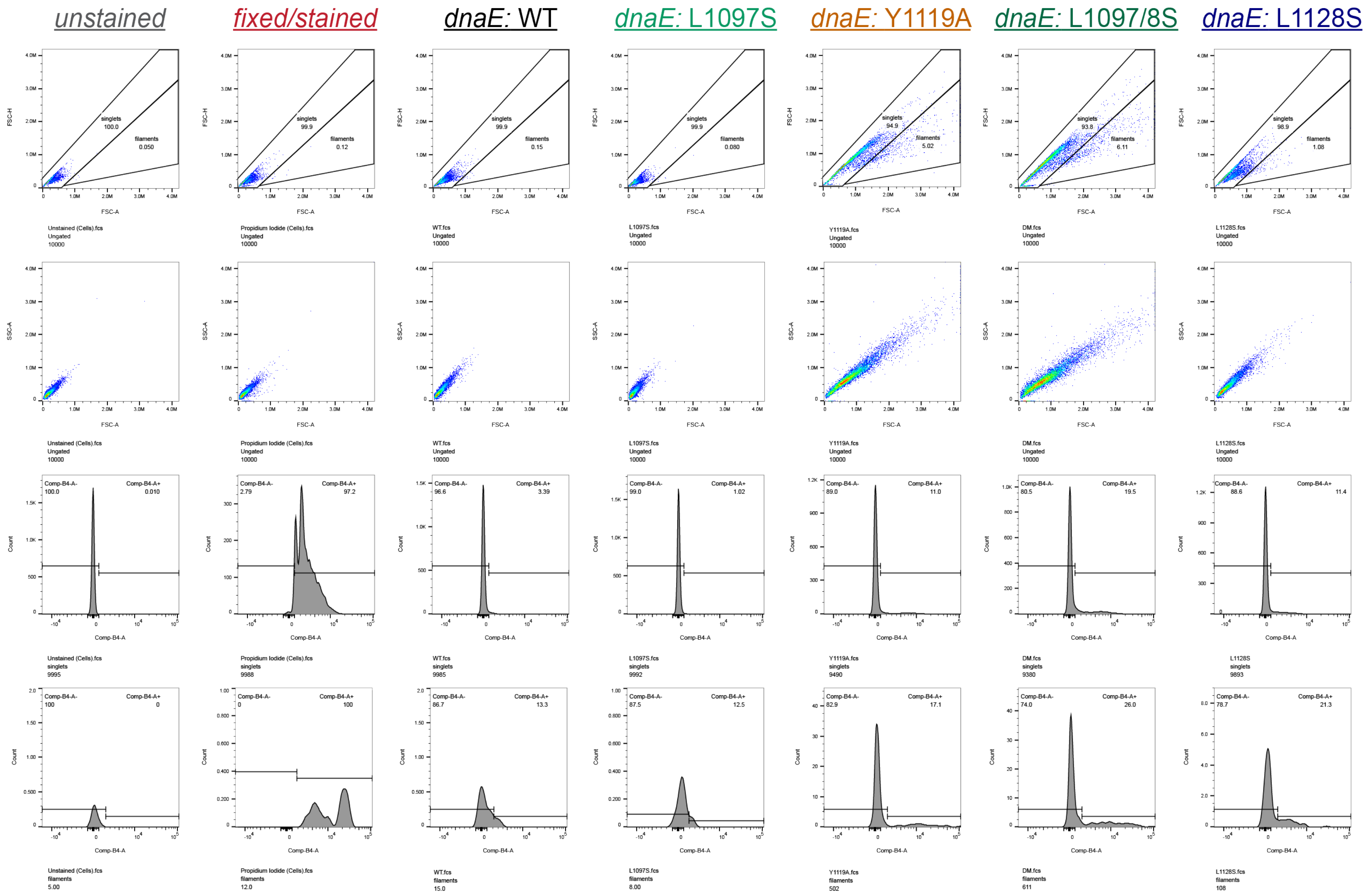

Supporting Figure S8: **FACS gating live/dead cell analysis of *dnaE* strains**. Stationary phase cells of *dnaE*:mut and WT strains stained with propidium iodide (PI) and analyzed by flow cytometry of 10,000 events (cells) total. A scatter plot of FSC-A (forward scatter area) versus FSC-H (forward scatter height) cells is shown, and the gating of singlet and filamented cell areas is defined (1st row). Also shown is a scatter plot of the FSC (forward scatter) versus SSC (side scatter) distribution of cells (2nd row). Both scatter plots indicate cell density by color, from highest to lowest; the cell concentration is shown in red, yellow, green, and then blue. The histograms show gated singlet (3rd row) and filamented (4th row) cells using unstained (neg control) and fixed/stained (pos control) populations as boundaries for live and dead cells.

References

1. Douma, L. G. (2016) Mechanism of sliding clamp loading by the *Escherichia coli* clamp loader complexes dissertation, University of Florida
2. Naufer, M. N., Murison, D. A., Rouzina, I., Beuning, P. J., and Williams, M. C. (2017) Single-molecule mechanochemical characterization of *E. coli* Pol III core catalytic activity. *Protein Sci.* **26**, 1413-1426
3. Tondnevis, F., Weiss, T. M., Matsui, T., Bloom, L. B., and McKenna, R. (2016) Solution structure of an "open" *E. coli* Pol III clamp loader sliding clamp complex. *J. Struct. Biol.* **194**, 272-281
4. Wieczorek, A., Downey, C. D., Dallmann, H. G., and McHenry, C. S. (2010) Only one ATP-binding DnaX subunit is required for initiation complex formation by the *Escherichia coli* DNA polymerase III holoenzyme. *J. Biol. Chem.* **285**, 29049-29053
5. Johanson, K. O., Haynes, T. E., and McHenry, C. S. (1986) Chemical characterization and purification of the beta subunit of the DNA polymerase III holoenzyme from an overproducing strain. *J. Biol. Chem.* **261**, 11460-11465
6. Carney, S. M., Gomathinayagam, S., Leuba, S. H., and Trakselis, M. A. (2017) Bacterial DnaB helicase interacts with the excluded strand to regulate unwinding. *J. Biol. Chem.* **292**, 19001-19012
7. Behrmann, M. S., Perera, H. M., Hoang, J. M., Venkat, T. A., Visser, B. J., Bates, D. *et al.* (2021) Targeted chromosomal *Escherichia coli:dnaB* exterior surface residues regulate DNA helicase behavior to maintain genomic stability and organismal fitness. *PLoS genetics* **17**, e1009886
8. Griep, M. A., and McHenry, C. S. (1989) Glutamate overcomes the salt inhibition of DNA polymerase III holoenzyme. *J. Biol. Chem.* **264**, 11294-11301
9. Monachino, E., Ghodke, H., Spinks, R. R., Hoatson, B. S., Jergic, S., Xu, Z. Q. *et al.* (2018) Design of DNA rolling-circle templates with controlled fork topology to study mechanisms of DNA replication. *Anal. Biochem.* **557**, 42-45
10. Chen, S. H., Byrne, R. T., Wood, E. A., and Cox, M. M. (2015) *Escherichia coli radD* (*yejH*) gene: A novel function involved in radiation resistance and double-strand break repair. *Mol. Microbiol.* **95**, 754-768
